# Relationship between myocardial oxygenation and blood pressure: Experimental validation using oxygenation-sensitive cardiovascular magnetic resonance

**DOI:** 10.1371/journal.pone.0210098

**Published:** 2019-01-16

**Authors:** Dominik P. Guensch, Kady Fischer, Christof Jung, Samuel Hurni, Bernhard M. Winkler, Bernd Jung, Andreas P. Vogt, Balthasar Eberle

**Affiliations:** 1 Department of Anaesthesiology and Pain Medicine, Inselspital, Bern University Hospital, University of Bern, Bern, Switzerland; 2 Institute for Diagnostic, Interventional and Paediatric Radiology, Inselspital, Bern University Hospital, University of Bern, Bern, Switzerland; 3 McGill University Health Centre, Montreal, QC, Canada; 4 Department of Cardiovascular Surgery, Inselspital, Bern University Hospital, University of Bern, Bern, Switzerland; Scuola Superiore Sant'Anna, ITALY

## Abstract

**Background:**

The relationship between mean arterial pressure (MAP) and coronary blood flow is well described. There is autoregulation within a MAP range of 60 to 140 mmHg providing near constant coronary blood flow. Outside these limits flow becomes pressure-dependent. So far, response of myocardial oxygenation to changes in pressure and flow has been more difficult to assess. While established techniques mostly require invasive approaches, Oxygenation-Sensitive (OS) Cardiovascular Magnetic Resonance (CMR) is a technique that can non-invasively assess changes in myocardial tissue oxygenation. The purpose of this study was to follow myocardial oxygenation over a wide range of blood pressure variation within and outside known coronary autoregulatory limits using OS-CMR, and to relate these data to coronary hemodynamics.

**Methods:**

Ten anaesthetized swine (German Large White) underwent left-sided thoracotomy and attachment of a perivascular flow probe to the proximal left anterior descending (LAD) coronary artery for continuous measurement of blood flow (Q_LAD_). Thereafter, animals were transferred into a 3T MRI scanner. Mean arterial pressure (MAP) was varied in 10–15 mmHg steps by administering alpha_1_-receptor agents phenylephrine or urapidil. For each MAP level, OS-CMR images as well as arterial and coronary sinus blood gas samples were obtained simultaneously during brief periods of apnea. Relative changes (Δ) of coronary sinus oxygen saturation (ScsO_2_), oxygen delivery (DO_2_) and demand (MVO_2_), extraction ratio (O_2_ER) and excess (Ω) from respective reference levels at a MAP of 70 mmHg were determined and were compared to %change in OS-signal intensity (OS-SI) in simultaneously acquired OS-CMR images.

**Results:**

Q_LAD_ response indicated autoregulation between MAP levels of 52 mmHg (lower limit) and127 mmHg (upper limit). OS-CMR revealed a global myocardial oxygenation deficit occurring below the lower autoregulation limit, with the nadir of OS-SI at -9.0%. With MAP values surpassing 70 mmHg, relative OS-SI increased to a maximum of +10.6%. Consistent with this, ΔScsO_2_, ΔDO_2_, ΔMVO_2_, ΔO_2_ER and ΔΩ responses indicated increasing mismatch of oxygenation balance outside the autoregulated zone. Changes in global OS-CMR were significantly correlated with all of these parameters (p≤0.02) except with ΔMVO_2_.

**Conclusion:**

OS-CMR offers a novel and non-invasive route to evaluate the effects of blood pressure variations, as well as of cardiovascular drugs and interventions, on global and regional myocardial oxygenation, as demonstrated in a porcine model. OS-CMR identified mismatch of O_2_ supply and demand below the lower limit of coronary autoregulation. Vasopressor induced acute hypertension did not compromise myocardial oxygenation in healthy hearts despite increased cardiac workload and O_2_ demand. The clinical usefulness of OS-CMR remains to be established.

## Introduction

### Coronary autoregulation

Myocardial blood flow in humans delivers approximately 70-80ml/min/100g myocardial tissue at rest. Coronary flow reserve can increase myocardial blood flow up to 3-5-fold from resting conditions [1–3]. Vascular autoregulation is characteristic for vital organs such as heart and brain, ensuring adequate and near constant tissue blood flow over a wide range of blood pressure [4]. Thus, blood pressure variation within autoregulatory limits should not compromise delivery of O_2_ and nutrients. The main mechanism of blood pressure-dependent regulation of blood flow has been proposed in 1902 by Bayliss [5], and is known since as the Bayliss effect or myogenic control of vascular tone [6]. In healthy humans, coronary autoregulation has been reported to be effective within a range of mean arterial pressures (MAP) between approximately 60 and 140mmHg. Such limits may vary with different pathologies, and higher perfusion pressures may be required to maintain constant blood flow [7]. Especially in the presence of a fixed coronary stenosis or of overriding coronary vasodilation, blood flow becomes pressure dependent [8].

Coronary perfusion of the left ventricular myocardium mainly occurs during diastole. Hence, an increase in aortic diastolic pressure and a longer diastolic time will improve perfusion. When MAP increases beyond the upper autoregulatory limit, coronary blood flow is markedly increased and becomes pressure dependent. Arterial hypertension will also increase oxygen demand and may reduce subendocardial blood flow [9]. This can outweigh enhanced oxygen supply from coronary vasodilation. In fact such challenges have been shown to increase oxygen demand and myocardial oxygen extraction [2,7–10]. Thus, severe hypertension may uncouple oxygen demand from supply and may compromise myocardial oxygenation. This effect has traditionally been assessed by calculating oxygen supply and demand from invasive blood flow measurements and oximetry of the in- and out-flux blood. Data based on direct measurement of myocardial tissue oxygenation are scarce.

### Conventional measures of oxygen supply and demand

Myocardial oxygenation depends on the balance of local oxygen supply (DO_2_) and demand (MVO_2_) [2,9,11,12]. Global myocardial oxygenation balance can be assessed by measuring arterial and coronary sinus haemoglobin, its oxygen saturation (ScsO_2_) and content [13]. ScsO_2_ is obtained invasively, e.g. via a surgically or fluoroscopically placed catheter in the coronary sinus. Oxygen extraction ratio (O_2_ER) is another parameter to describe the relationship between oxygen supply and demand [14].

DO_2_ and MVO_2_ are determined by obtaining haemoglobin concentration, blood gas analysis and oximetric status from affluent and effluent blood together with blood flow measurement [15]. This global approach has the limitation that there is no information about regional supply-demand mismatch. Especially in coronary artery disease, global estimates are insensitive to insufficient blood flow in specific myocardial territories. Regionally resolved blood flow and oxygen content measurement would be required to assess regional oxygenation balance, which are clinically not feasible so far. Direct measurement of tissue oxygen tension or haemoglobin saturation have been proposed and would be preferable, but such methods are also invasive or require probes which are restricted to experimental settings only, for reasons of toxicity [16,17].

### Assessment of myocardial oxygenation using cardiovascular magnetic resonance

Oxygenation Sensitive (OS) Cardiovascular Magnetic Resonance (CMR) is a non-invasive technique to map and to follow myocardial oxygenation changes. It uses the Blood Oxygen Level-dependent (BOLD-) effect to generate a contrast in MRI sequences susceptible to this effect. Pauling proposed in 1936 that deoxygenated haemoglobin (dHb) has magnetic properties differing from those in the oxygenated (HbO_2_) state [18]. Ogawa was the first to use this mechanism in the field of MRI imaging of the brain and proposed that the paramagnetic effects of dHb disturb magnetic field homogeneity on a molecular level [19]. This effect accelerates transverse magnetic relaxation through spin-spin interactions in T2- or T2*-sensitive MRI sequences, which decreases signal intensity (SI) in the resulting images. The diamagnetic HbO_2_ instead results in weak stabilization of the magnetic field, with no change in SI. While BOLD contrast effects have been utilized in functional MRI scans for long, exploitation of the same effect in the heart for OS-CMR has been developed only more recently [20]. Today, MR sequences have gained enough spatial and temporal resolution to allow introduction of OS-CMR to human diagnostics. Signal attenuation in OS-CMR images originates in the compartment of the post-capillary myocardial venules [21,22]. Mechanisms that increase dHb concentration, such as diminished oxygen supply (low SaO_2_, decreased blood flow) or increased oxygen extraction (e.g. during increased workload) attenuate local signal intensity (SI). Factors which reduce dHb concentration, like blood flow augmentation or reduction of oxygen demand (luxury perfusion), will enhance OS-signal intensity (SI) and produce regional hyperintensity [23]. Better regional oxygenation will thus be reflected by ipsi-regional increased SI when compared to a reference image. In contrast to ScsO_2_ [24], OS-CMR is capable to detect also regional oxygenation changes, with a resolution given by the size of the imaged voxels (defined by in-plane resolution and slice thickness, e.g. voxels of 2x2x10mm). SI is not an absolute measurement but must be interpreted as SI change, following a stimulus, in relation to a reference SI in the same region of interest. The derived proportional change in SI will represent a relative increase or decrease in myocardial tissue oxygenation in reference to the condition during which the reference image was acquired. Such SI changes at OS-CMR allow to follow changes in local post-capillary SO_2_, and hence, oxygen balance on a millimetre scale. Meanwhile, OS-CMR has been validated against other diagnostic modalities such as Positron Emission Tomography (PET)[25], Fractional Flow Reserve [26,27], and myocardial energetic indices in humans [28]. Experimental studies have established more fundamental relationships of OS-CMR change to invasively measured parameters such as arterial oxygen [29,30] and carbon dioxide partial pressure [31–33], coronary artery [34] and coronary sinus blood flow [24], labelled microsphere measurements, [35], coronary sinus oxymetriy [24] and haematocrit [36].

The primary aim of this study was to describe the myocardial oxygenation response to arterial pressure variation using OS-CMR, as elicited by phenylephrine and urapidil titration in an animal model. Secondary goals were to explore relationships between OS-CMR data and concurrent coronary blood flow and oximetry measurements.

## Methods

### Animal preparation

With Cantonal Animal Care Committee approval, fifteen adolescent German landrace swine (29.9±2.2kg) were used in this study. Prior to experiments, the animals were housed at the University Veterinary Facilities for 48h for acclimatization and to assess their general health. Two to four hours prior to the experiments they were transferred into the animal surgery facilities. Fasted for 6h and with free access to water until 2 hours prior the experiments, animals were premedicated with 20 mg/kg ketamine and 2 mg/kg xylazine intra-muscularly. Following IV induction with 10 mg of midazolam and 1 mg atropine, the trachea was intubated. Ventilator settings were a tidal volume of 6–8 ml/kg, positive end-expiratory pressure of 5 mbar, an inspiratory oxygen fraction (FiO_2_) of 0.4 and a respiratory rate of 15-20/minute to achieve end-tidal CO_2_ partial pressures of 35–40 mmHg. Anaesthesia was maintained with continuous i.v. fentanyl (5–30μg/kg/hour) and propofol (4–8 mg/kg/hour). A 4-French (F) sheath was placed into a femoral artery for continuous invasive pressure and blood gas measurement. A 6F sheath inserted into a femoral vein was used to administer fluids (10-15ml/kg/h lactated Ringer’s Solution) and medication. The right jugular vein was cannulated with a 11F sheath to insert a catheter in the coronary sinus, whose correct position was confirmed by tactile feedback from the cardiac surgeon. Continuous monitoring included pulse oximetry (SpO_2_) and capnography, standard 3-lead electrocardiogram (ECG) and invasive arterial blood pressure. After i.v. anti-thrombotic (heparin 5000 IU) and anti-arrhythmic (75 mg amiodarone) prophylaxis, a left lateral thoracotomy was performed. An MR-compatible perivascular flow probe (Transit-time perivascular flowmeter, Type TS420, Transonic Systems Inc., Ithaca, NY, USA) was then attached onto the proximal left anterior descending coronary artery (LAD).

### Experimental protocol

Animals were transferred to a clinical 3 Tesla MRI (Magnetom Trio, Siemens AG, Erlangen, Germany), and placed in supine position. A mean arterial pressure (MAP) of 70mmHg (±5mmHg) was defined as baseline status for the study and all subsequent analyses, assumed to be within coronary autoregulatory limits at normoxaemia and normocapnia. A cine short-axis stack was acquired for analysis of baseline left ventricular (LV) function. As the intervention, MAP was manipulated in 10-15mmHg increments by dose variation of the α_1_-receptor agonist phenylephrine (16–660μg/min per infusion pump) or by administration of the α_1_-receptor antagonist urapidil (5-10mg repetitive i.v.) as required by each individual subject. For reasons of hemodynamic stability, MAP level sequence was not randomized but hypotensive MAP levels were targeted first. Urapidil was administered until blood pressure could not be further lowered, then phenylephrine dose was increased until the ceiling effect precluded any further increase in MAP. After reaching a predefined MAP level, hemodynamics were allowed to stabilize for at least 30 seconds. Data acquisition started after fluctuation in LAD blood flow (*Q*_*LAD*_) had subsided. OS-CMR scans were performed in two short axis slices downstream to the blood flow probe (imaging parameters and analysis see S1 File). Blood gas samples were obtained simultaneously from arterial and coronary sinus catheters after the scan had commenced. During pharmacological interventions, *Q*_*LAD*_, arterial blood pressure and heart rate were recorded continuously. At the end of experiments, animals were euthanized with i.v. overdose of propofol and potassium chloride.

### Calculations derived from blood gases and myocardial blood flow

Arterial (CaO_2_) and coronary sinus (CcsO_2_) oxygen content, DO_2_, MVO_2_, difference in DO_2_ and MVO_2_, oxygen extraction ratio (O_2_ER), oxygen excess (Ω) and myocardial lactate production were calculated as outlined in S1 File.

### Image and statistical analysis

To analyse myocardial oxygenation changes at OS-CMR for each level of MAP, signal intensity (SI) of each OS-CMR image was obtained, requiring consensus of two readers, by drawing endocardial and epicardial contours at end-systole. This was primarily reported for the global myocardial value. The LAD specific territory was also analysed for comparison with *MAP*, *Q*_*LAD*_ and the derived *DO*_*2*_. This was obtained by automatic segmentation (segments 1, 2, 7 and 8) by the imaging software according to the AHA 17 segment model[37]. Change of within-subject OS-CMR signal intensity (OS-SI) and of oximetry data was reported as relative change (percent) from the baseline level (MAP of 70mmHg).

Data was assessed for normality using a D’Agostino-Pearson test. Results are given as mean ± standard deviation. Response of dependent variables to changes in MAP was analysed for linearity or curvi-linearity, and the model with the smallest sum-of-squares was chosen as best fit. Analysis accounted for multiple within-subject comparisons. For additional statistical modelling, linearity was assumed. To investigate the linear relationship between relative changes from baseline in variables DO_2_, MVO_2_, O_2_ER and ScsO_2_ to both myocardial oxygenation and MAP, a within-subjects correlation coefficient was determined to account for repeated measurements within each animal[38], using the ‘rmcorr’ package with *R* software (version 3.4.4). The primary statistical outcome described the relationship between global OS-CMR changes and MAP. Further statistical analysis used Graphpad Prism (version 7, Graphpad Inc. San Diego, California, USA). Results were considered significant at *p*<0.05 (two-tailed).

### Ethics statement

This study was conducted in accordance with national animal care regulations and approved by the Veterinary Services at the Department of Agriculture and Nature of the Canton Bern, Switzerland [#BE 103/14].

## Results

Imaging lasted between 60–256 minutes. Ten animals provided full datasets. Two more were excluded due to experimental hardware problems. Another two had episodes of ventricular fibrillation, and one suffered from peri-myocarditis as discovered during surgery. All animals with full datasets had an LV function normal for swine (Table 1)[39]. Animals did not show bradycardic responses to induced hypertension. Neither treatment with urapidil nor phenylephrine had an effect on heart rate (r = -0.109, p = 0.289). The data passed normality distribution tests.

**Table 1:** Baseline left-ventricular (LV) function parameters assessed by CMR.

Parameter	Mean ± SD
EDV (ml)	57 ±11
ESV (ml)	27 ±10
SV (ml)	29±5
EF (ml)	53±10
CO (L/min)	2.4±0.4
HR (bpm)	80±16
LV Mass (g)	64±10

End-diastolic volume (EDV), end-systolic volume (ESV), stroke volume (SV), cardiac output (CO), heart rate (HR), LV mass.

### Coronary artery blood flow and limits of autoregulation

**Fig 1:**
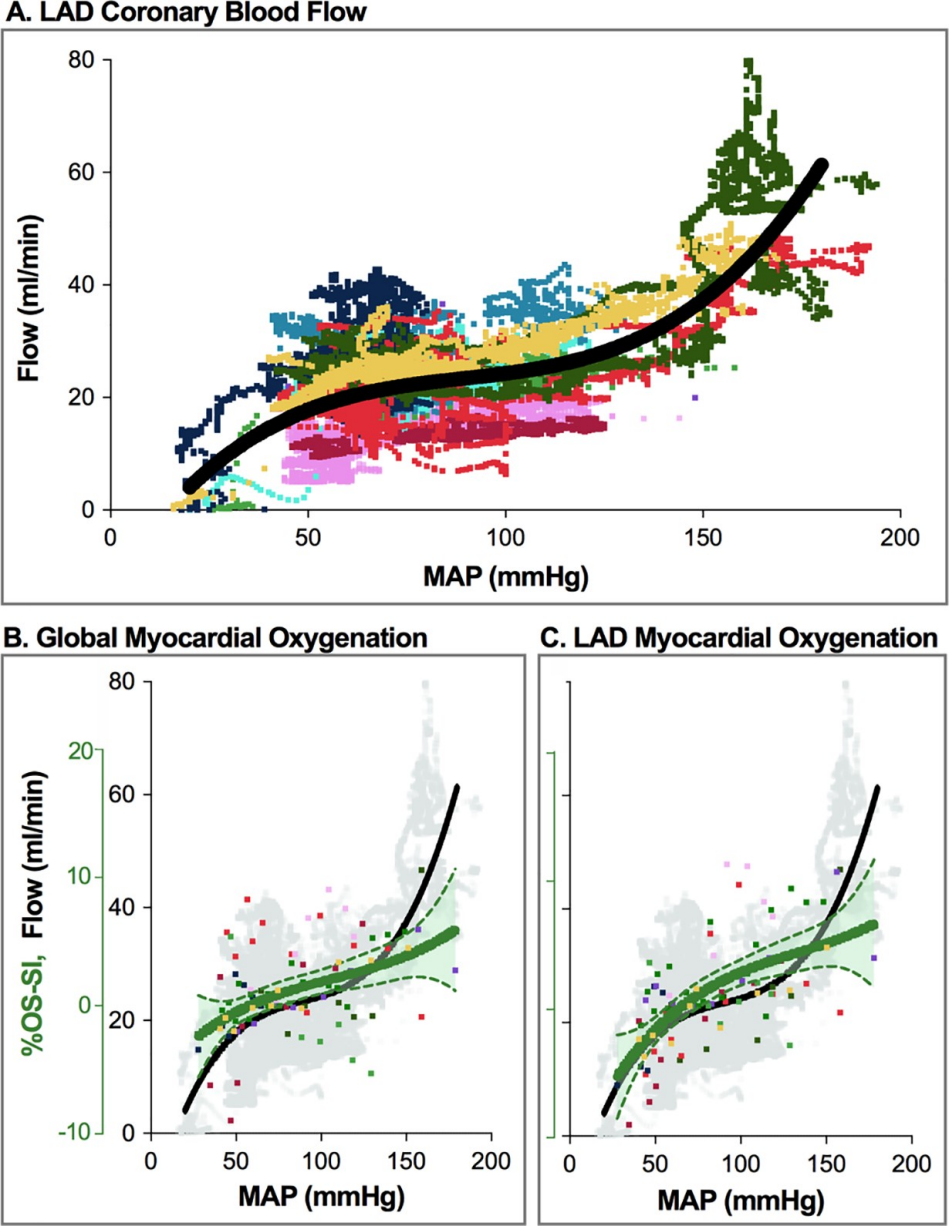
LAD blood flow and myocardial oxygenation across the tested range of mean arterial pressure. **A**. Absolute LAD blood flow (ml/min) (n = 10 animals, each represented by a different colour). There is a plateau indicating autoregulation between MAP of 52–127 mmHg, with steeper slopes outside these limits (n = 55’368 data points). **B**. Global oxygenation-sensitive signal intensity (%-OS-SI) responds non-linearly to increasing MAP (n = 94). Above a MAP of 160 mmHg there was larger variation in OS-SI response. **C**. Regional signal intensity response is shown for myocardial territories subtended by the LAD.

LAD flow (Q_LAD_) response to MAP changes was best explained by a curvi-linear model (Fig 1). Since Q_LAD_ was recorded throughout the entire experiment, 55’368 data points were available with MAP ranging between 194mmHg and 16mmHg. These extremes were associated with a Q_LAD_ of 80ml/min and 0ml/min, respectively. Stable MAP levels during periods of CMR and oximetry acquisition ranged from 28-196mmHg and corresponded to Q_LAD_ ranging between 8 and 64ml/min.

The center of the fitted pressure-flow relationship (MAP-Q_LAD_) was determined at MAP of 89mmHg (see Figure A in S1 File for detailed explanation of fitting and calculation). Within this autoregulatory range, Q_LAD_ varied 19±1% (19.0±0.3ml/min) and 23±1% (28.0±0.5ml/min) from Q_LAD_ at the centre of the autoregulatory range (23ml/min).

Linear fitting from continuous measurements in ten animals indicated good correlation between absolute Q_LAD_ and MAP data (r = 0.750, p<0.001). The flow response [%] compared to baseline flow at 70mmHg MAP revealed a similar correlation to MAP (r = 0.772, p<0.001). Relationships of MAP and flow for defined blood pressure levels, where blood gas measurements and OS-CMR scans occurred, can be seen in Table 2.

**Table 2:** Within subject correlation coefficients.

	MAP		%OS-SI_Global_		%OS-SI_LAD_	
	r	*p-value*	r	*p-value*	r	*p-value*
%-OS-SI_Global_	0.326	0.002*	-	-	-	-
%-OS-SI_LAD_	0.604	<0.001*	-	-	-	-
%-Flow (Q_LAD_)	0.699	<0.001*	0.452	<0.001*	0.651	<0.001*
ΔDO_2_	0.784	<0.001*	0.361	0.002*	0.586	<0.001*
ΔMvO_2_	0.820	<0.001*	0.228	0.082	-	-
ΔDO_2_ - ΔMvO_2_	0.795	<0.001*	0.433	<0.001*	-	-
ΔScsO_2_	0.301	0.012*	0.402	0.001*	-	-
ΔΩ	0.335	0.005*	0.290	0.024*	-	-
ΔO_2_er	-0.342	0.018*	-0.290	0.024*	-	-

All measures of cardiac oxygenation and LAD blood flow were linearly associated with change in MAP. Invasively derived measures were linearly associated with global OS-CMR response (%-OS-SI_Global_) except ΔMvO_2_.

The OS-CMR response of the LAD specific territory (%-OS-SI_LAD_) was compared to MAP, Q_LAD_ and ΔDO_2_ only. r = within-subjects Pearson’s co-efficient.

*p<0.05.

### Balance of myocardial oxygen supply and demand derived from invasive measurements

**Fig 2:**
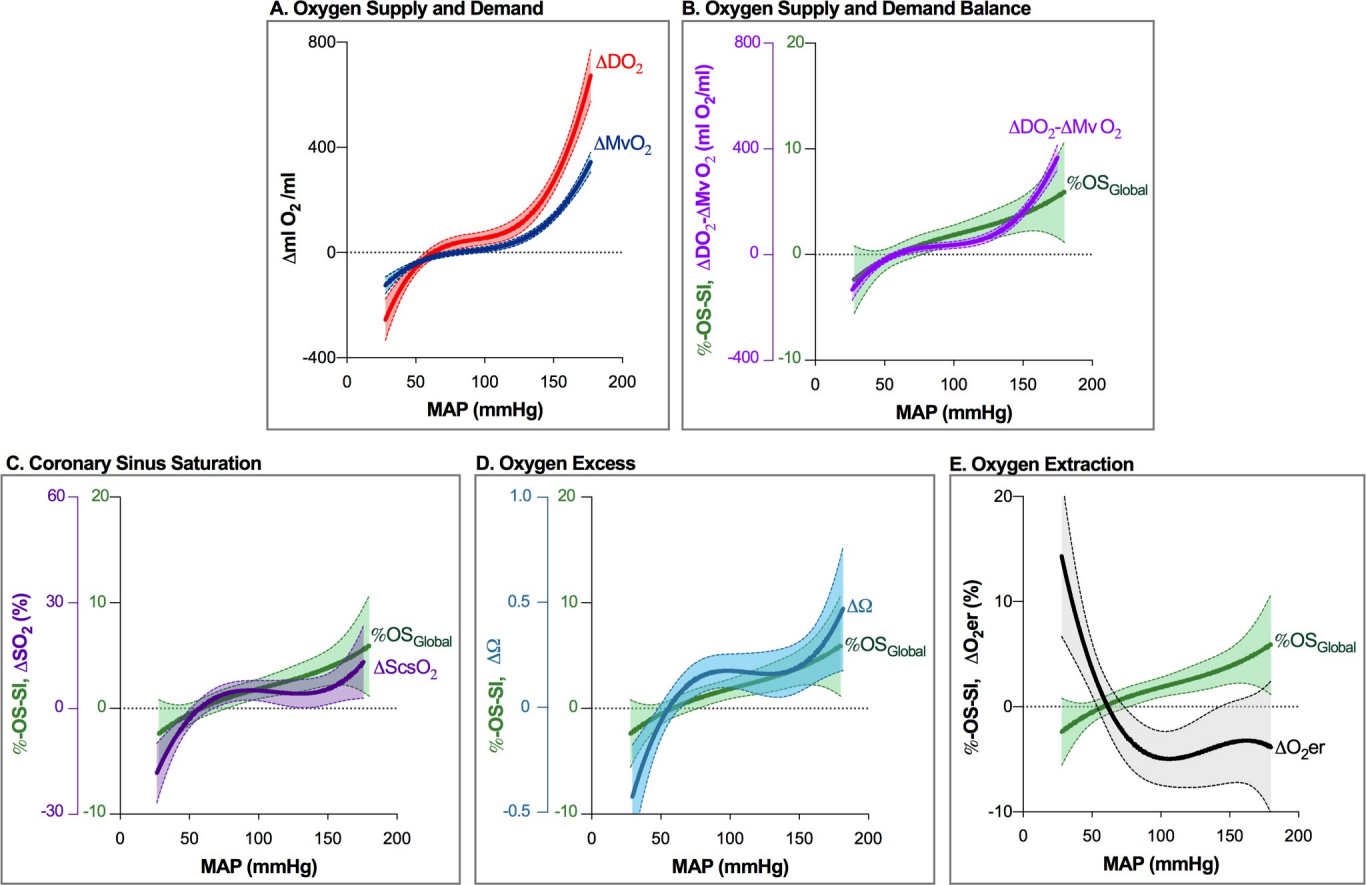
Non-linear response of descriptors of myocardial oxygenation balance to pharmacologically induced blood pressure changes. **A.** Response of oxygen delivery (ΔDO_2_, red, n = 88) and myocardial oxygen consumption (ΔMVO_2_, dark blue, n = 74) relative to their baseline (arbitrarily set at a MAP of 70mmHg). Both, ΔDO2 and ΔMVO_2_ reveal an autoregulatory plateau and steep slopes beyond autoregulatory limits. Within the autoregulation zone, DO_2_ consistently surpasses MVO_2_, whereas there is mismatch between DO_2_ and MVO_2_ outside autoregulatory limits. At hypotension, ΔMVO_2_ is no more matched by ΔDO_2_, while there is redundant oxygen supply at hypertensive MAP beyond 130 mmHg. **B**. Curvilinear response to MAP variation of arterio-venous difference (ΔDO_2_ - ΔMVO_2_; purple n = 74) and myocardial oxygenation response (%OS-SI; green, n = 94). Note upslope beyond autoregulatory limits. **C**. Curvilinear response to MAP of coronary sinus oxygen saturation (ScsO_2_, purple, n = 75) and **D.** oxygen excess Ω (blue, n = 75). **E.** MAP-associated OS-SI rise is accompanied by falling coronary blood oxygen extraction ratio (O_2_eER, black, n = 75), due to improving DO_2_. x-axis intercept of fitted curves is at a MAP of 50-60mmHg. This indicates the lower limit of autoregulation and is consistent with calculated limits (see S1 File). Line: fitted mean of non-linear regression; shaded area: 95% confidence interval.

Results of the univariate analysis for changes in global oxygenation balance parameters with MAP variation are listed in Table 2. Regional analysis showed significant correlation between %OS-SI in LAD perfused territories and LAD blood flow (r = 0.651, p<0.001). Fig 2 depicts the moderate increase of DO_2_ and MVO_2_ within the autoregulation zone, and a steep rise beyond its upper limit, where ΔDO_2_ increased more than ΔMvO_2_. Towards hypotension, oxygen supply and demand became progressively mismatched, i.e. ΔDO_2_ decreased more than ΔMVO_2_. MAP dependent changes in ΔScsO_2_, ΔΩ and ΔO_2_ER levelled in a MAP range between approximately 90 and 180mmHg. MAP below 80 mmHg resulted in steeply rising ΔO_2_ER and falling ΔScsO_2_ and ΔΩ. Absolute measurements are given in Figure B in S1 File. No increase in myocardial lactate production was observed in relation to MAP.

### Myocardial tissue oxygenation assessed with OS-CMR

**Fig 3:**
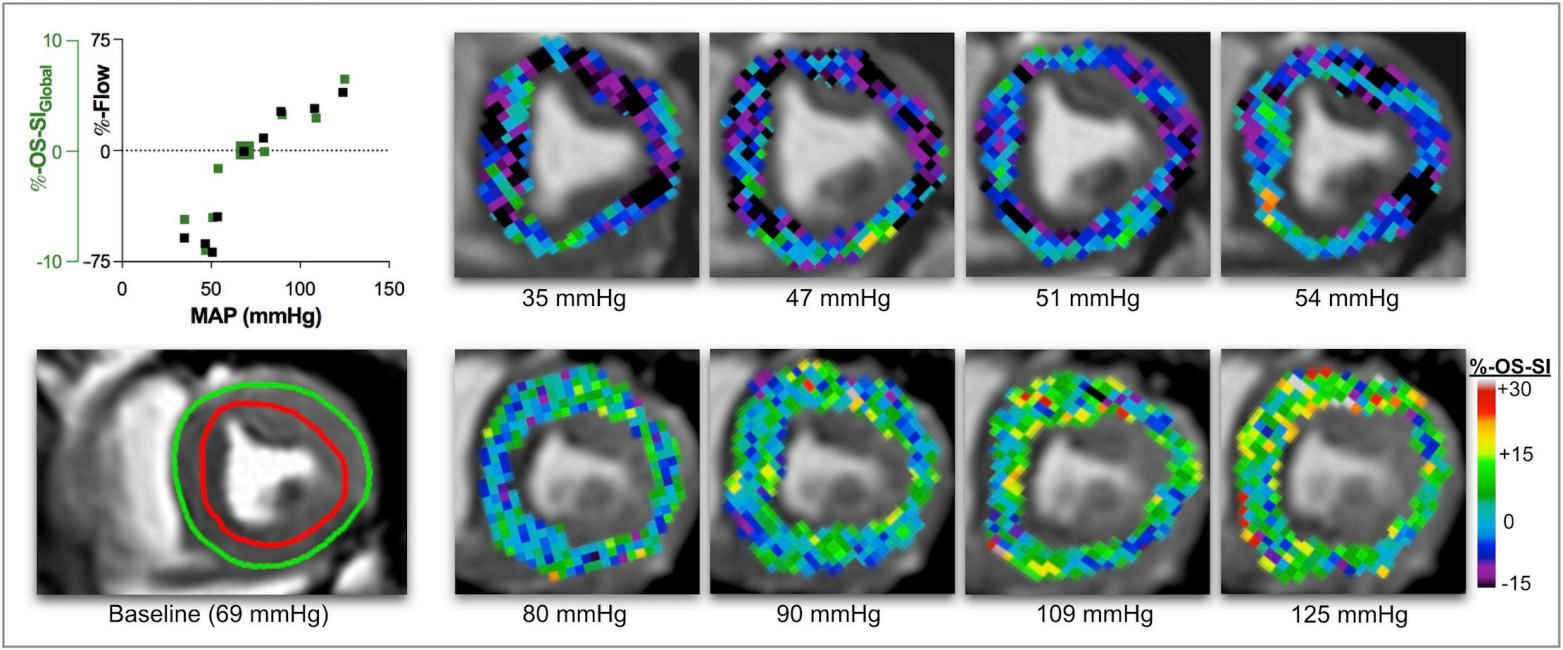
Myocardial oxygenation response at OS-CMR. In this individual animal, colour maps represent percent change of signal intensity (OS-SI) in systolic OS-CMR images in comparison to baseline. At nadir MAP of 35 mmHg there is relative de-oxygenation of -6.2% (black, purple). Relative OS-SI increases to a maximum of +6.4% at a MAP of 125mmHg (green, yellow). In this animal relative OS-SI responds in close association with blood flow (top left). Colour maps are generated for visual representation only (neurolens.org). The bottom left panel demonstrates analysed myocardium (on the raw OS-CMR image, between green and red contour) in a systolic mid-papillary short-axis slice at a baseline MAP of 69mmHg.

OS-CMR image quality was acceptable at a 9.1% exclusion rate. Of the 103 levels scanned during the series, nine data sets from five animals were excluded because of poor image quality, resulting in 94 levels available for analysis. OS-SI derived myocardial oxygenation changes of all animals across MAP levels are given in Fig 1B and 1C and Table 2. Global analysis (OS-SI_Global_) gave a range across all MAP levels of 18.6% (-9.0% to +10.6%). Regional LAD specific range (OS-SI_LAD_) was 24.2% (-9.3% to +14.9%). Myocardial oxygenation decreased when MAP dropped below a lower autoregulation limit at approximately 50-60mmHg. The subtraction map (Fig 3) shows myocardial oxygenation changing during a representative experiment, when MAP departed from the 70mmHg baseline. The colour overlay demonstrates that myocardial oxygenation increased when MAP rises above 70mmHg, while it progressively deteriorates with hypotension.

Variation from baseline of ΔScsO_2_ ranged from -31 to 21%, and of ΔO_2_ER from -9.6 to 19.3%. The fitted curve of OS-SI_Global_ changes (Fig 2B) also revealed a curvilinear response to MAP manipulation, with reciprocal behavior of O_2_ER. (Fig 2A). Global myocardial oxygenation parallels flow within autoregulatory limits but uncouples outside autoregulation limits (Fig 1B), affecting ΔScsO_2_ and ΔΩ. The LAD oxygenation curve (Fig 1C) matches MAP better below the lower autoregulation limit than OS-SI_Global_.

## Discussion

This study relates myocardial oxygenation changes, as assessed with non-invasive Oxygenation-Sensitive CMR imaging, to established parameters such as DO_2_, MvO_2_, ScsO_2_, Ω and O_2_ER and MAP. Myocardial oxygenation data determined non-invasively using OS-CMR correlated systematically with physiological parameters of coronary O_2_ supply and myocardial demand. Notably, similar to established invasive parameters, OS-CMR was able to identify compromise of myocardial oxygenation balance below the lower autoregulation boundary, as indicated by a decrease in OS-SI. All parameters also identified relative luxury perfusion at high mean arterial pressures.

### Effects of phenylephrine and urapidil on coronary artery motion

For manipulating MAP in this model we preferred phenylephrine over noradrenaline. Although norepinephrine primarily binds to alpha-1 receptors on smooth muscles, induces vasoconstriction and may elicit baroreceptor-mediated bradycardia, it also has beta-adrenergic effects and may increase heart rate with higher concentrations. In preliminary experiments to this study in swine, norepinephrine administration resulted in a marked increase in heart rate, which would have confounded DO_2_ and MVO_2_. Importantly, in a porcine animal model it has been demonstrated to also elicit a direct vasoconstrictive effect on coronary arteries [40]. Phenylephrine is a synthetic selective alpha-1-receptor agonist. It has been shown in canine, porcine and human studies to exert no or minimal vasoconstricting effects on coronary arteries [40–42]. The characteristics of the MAP-Q_LAD_ relationship in our study indicated that the use of phenylephrine had no relevant effect on coronary vasomotor tone itself and thus had no confounding effects. Also, urapidil did not induce relaxation of porcine coronary arteries according to data of Bopp et al [43]. Thus, direct effects on coronary vascular tone by the agents chosen for MAP manipulation appear unlikely in our experimental setup of pharmacologically induced systemic blood pressure changes.

### Impact of systemic blood pressure changes on myocardial perfusion

In healthy hearts, an acute rise of systemic blood pressure increases left ventricular afterload, myocardial workload but also coronary blood flow. Increasing myocardial blood flow enhances DO_2_ to match rising MVO_2_, compensating for higher myocardial workload [2]. However, myocardial O_2_ balance may become compromised when the rise of systemic arterial resistance increases LV myocardial workload so much that at very high MAP, MVO_2_ can exhaust DO_2_ and tissue oxygenation becomes critical [15]. A crucial factor for microvascular blood flow in the heart is extravascular pressure exerted on the microvasculature by myocardial wall tension [3,44]. Wall tension depends on end-diastolic filling pressure, wall thickness, ventricular dimension, heart rate and contractility [2,3]. The left ventricular pressure during systole has to surpass the aortic diastolic blood pressure in order to eject the blood through the aortic valve. In contrast to the right ventricle, systolic intraventricular pressure in the LV is higher than the pressure in the microvessels (20-30mmHg) in the healthy individual [6], which leads to a cessation of myocardial blood flow in systole especially in sub-endocardium [3]. With increasing systemic blood pressure, microvascular blood flow and oxygenation may thus become gradually compromised [9]. Although the sub-epicardial zones are perfused during diastole and systole, higher pressures and increased inotropy may affect these layers during systolic contraction alike. Since especially sub-endocardial blood flow occurs in diastole, an increase in heart rate with a corresponding shortening of the diastolic phase may further compromise blood flow. Significant heart rate changes during blood pressure manipulation could be avoided successfully in this study.

### Myocardial oxygen balance assessed by invasively obtained parameters

As seen in Fig 2, with increasing MAP levels DO_2_ already increases discretely more than MVO_2_ within the autoregulation zone, but even more pronounced beyond the upper autoregulation zone limit, indicating luxury perfusion. DO_2_ and MVO_2_ however are not direct measurements of myocardial oxygenation and are driven by calculation of blood gases and myocardial blood flow. Therefore, other factors influencing such variables will confound DO_2_ and MVO_2_ calculation, i.e. isolated changes in systemic blood gases due to changes in ventilation. Thus, some reviews question the utility and accuracy of these calculations [45,46]. Importantly, changes in systemic paO_2_ and paCO_2_ are known to affect coronary vascular tone. They may thus affect myocardial blood flow and deflect the steady state in metabolically coupled coronary blood flow. Increased paCO_2_ is a potent coronary arteriolar vasodilator [47], while an increase in paO_2_ is known to have vasoconstricting properties. These effects have also been investigated with OS-CMR, showing changes in myocardial oxygenation [30,31,34]. Thus, systemic blood gas changes may confound results in DO_2_ and MvO_2_ calculations.

Since DO_2_ increases more than MVO_2_ with rising MAP, indicating luxury perfusion, the excess flow Q is also factored into the MVO_2_ calculation. This may therefore lead to overestimation of MVO_2_. This should in fact lead to reduced oxygen extraction and stable or increasing levels in ScsO_2_ and consequently Ω. Especially with the steep increase in DO_2_ at very high MAP_,_ accuracy of MVO_2_ may be inaccurate and overestimated. Of note, with MAP decreasing, absolute DO_2_ is still greater than MVO_2_ (Figure B in S1 File), until their trends intercept at 35mmHg MAP. As ΔDO_2_ and ΔMVO_2_ indicate, blood pressures below the autoregulation zone tip the oxygenation balance when DO_2_ drops below MVO_2_, leading to a myocardial oxygenation deficit [15]. This is paralleled by the steep decrease of ΔScsO_2_, Ω, and increase of ΔO_2_er. With higher MAP both parameters level out. Of note, ΔO_2_er increases sharply at around 100mmHg with falling MAP. This does not fit the concept of a tightly regulated blood flow within the autoregulation zone to maintain stable oxygenation.

As a useful but indirect marker of whole-body oxygenation balance, mixed venous oxygen saturation (70–75%) measured in the pulmonary artery [48] cannot resolve organ-specific oxygen supply-demand mismatch [49]. In analogy, the coronary sinus drains venous blood with a baseline saturation of approximately 30–40% from the heart, reflectant of nearly maximum oxygen extraction from coronary influx by myocardium (Figure B in S1 File). Coronary sinus blood oximetry therefore does not allow assessment of regional supply-demand mismatch, either. Any method that can reliably and non-invasively detect such mismatch in defined myocardial territories would appear quite useful.

### Changes of myocardial oxygenation assessed by OS-CMR

OS-CMR can assess myocardial oxygenation directly and non-invasively. It does not rely on exogenous contrast or tracers accompanied by exposure to ionizing radiation. It has a sufficiently high spatial resolution to assess regional oxygenation [20]. There are different ways to assess this in myocardium using CMR. For instance, mapping techniques generate quantitative data which can be used to assess blood [50] and tissue oxygenation [23]. However, such sequences lack the temporal resolution, which is necessary for many experiments, and they are artifact-prone at higher magnetic field strengths. Our experimental setup required fast image acquisition, since maintenance of very hypertensive MAP levels for a sufficiently long period of time turned out to be challenging. Using an OS-cine sequence as reported in previous studies offered sufficient temporal resolution but came with the disadvantage that quantitative T2 or T2* values like in mapping techniques could not be generated [32,51]. However, changes in OS-SI were determined in reference to a baseline, and were compared to other parameters. Several studies used this technique before in experimental and clinical work [20],[24]. It has also been successfully used to interrogate the myocardium for regional deoxygenation in patients with coronary artery disease [27,52].

We assume that the inflection of the myocardial oxygenation curve below approximately 50–60 mmHg MAP in our study indicates the lower limit of coronary autoregulation. This is supported by invasive measurements. The association appeared even closer with LAD specific OS-CMR analysis. OS-CMR also confirms and reflects invasive data that myocardial oxygenation does not become compromised in healthy animals by induced hypertension. Analysis of correlations show that changes of OS-SI were best explained by proportional change of Q_LAD_, ΔDO_2_-ΔMVO_2_ and ΔScsO_2_. Similar findings have been reported in a validation study by Vöhringer et al. using OS-CMR to detect acute coronary artery stenosis. These authors compared OS-SI changes with flow and coronary sinus haemoglobin saturation, and found a linear correlation of OS-SI changes with ScsO_2_ changes [24]. OS-SI changes were also validated in vitro against venous blood oxygen saturation [36]. These and other studies have shown a close relationship between OS-SI and changes in blood oxygenation [29].

Above studies used sequence parameters and field strengths, which differed from our study. The modification of our MRI sequences accelerated image acquisition at the cost of reduced oxygenation sensitivity. A global myocardial oxygenation change of 18.6% at OS-CMR, respectively 24.2% for the LAD territory, means a significant deviation from baseline in capillaries under physiologic condition. However, OS data do not directly translate into coronary sinus saturation (total range 52.3%) but appear to closer match ΔO_2_er (total range 28.9%).

Spatial resolution of the sequence we used is, at 2x2x10mm, quite good. New advances using higher field strengths may offer new opportunities in oxygenation-sensitive imaging [53,54].

Our study shows, for instance, that OS-CMR has potential not only for evaluation of myocardial oxygenation in a variety of cardiac diseases. It may also be useful in cardiovascular drug trials, which investigate direct or indirect effects of such agents on myocardial oxygenation. OS-SI characteristics identified a similar lower autoregulation limit, intersected the x-axis at 50-60mmHg, and detected the oxygenation decline at lower blood pressure. LAD specific sub-analysis revealed a stronger loss of myocardial oxygenation below that limit, which appears consistent with decreasing blood flow (Fig 1C). Also, OS-CMR did not indicate compromised myocardial oxygenation at higher MAP. OS-CMR may also become an approach to revisit the effects of cardiovascular drugs. In the future, higher field strengths may provide us with even more layers of information.

### Limitations

This work has important limitations. First, it does not necessarily reflect human physiology, although autoregulation limits observed by us resemble the ones reported for humans [7]. Further, our autoregulation limits are similar to the ones reported for swine in the publication of Dick et al [8]. Anaesthetic agents could have attenuated the blood pressure response to phenylephrine and urapidil. There was quite a heterogeneity of the blood pressure. The time-frame was sufficient for obtaining OS images, but not of image stacks to look at concomitant changes in LV volumes, ejection fraction and cardiac output.

Also, we were only able to measure blood flow in the proximal LAD coronary artery but not the other two major vascular territories. This is also a limitation for the accuracy of DO_2_ and MVO_2_ calculations representing oxygenation balance of the entire myocardium.

OS-CMR analysis was performed at end-systole, as there is more myocardial tissue to analyse and usually fewer artefacts. Moreover, oxygen extraction is highest during systole in postcapillary haemoglobin, which therefore may best depict oxygenation balance in OS-CMR. Analysis during diastole would also be interesting since LV myocardial perfusion occurs mainly in this phase. Currently, the limited sampling of pixels renders this type of analysis still unprecise. More advanced OS-CMR sequences may overcome this problem in the future.

Last, in our model we used healthy animals to show that myocardial oxygenation is not compromised while increased coronary blood flow compensated for the increased myocardial workload. This may not be the case in hypertensive patients with hypertrophic or dilatative cardiomyopathies, coronary heart disease and heart failure. There, oxygen supply may fail to meet the increased oxygen demand.

## Conclusions

This study demonstrates that OS-CMR can be used to evaluate physiological effects or cardiovascular drug action on myocardial oxygenation in a non-invasive setting. In this healthy animal model, increasing mean arterial blood pressure did not compromise myocardial oxygenation despite increased cardiac workload and oxygen demand. OS-CMR was also able to identify insufficient oxygenation at blood pressures below the lower limit of autoregulation. OS-CMR, as a direct measure of myocardial oxygenation, may be superior to conventional invasive assessments of the myocardial oxygenation balance. More data is required to support this hypothesis.

## Supporting information

S1 File Supplementary methods in S1 File. a. Calculations derived from blood gases and myocardial blood flow
b. CMR sequences & image analysis
**Figure A in S1 File. Identification of the auto-regulation zone**.
The flow curve was generated by 55’368 data points from measurements of the left anterior descending coronary artery during manipulation of mean arterial blood pressures. A non-linear regression curve was fit to the data accounting for repeated measurements per subject. This resulting curve always demonstrates a positive slope at any MAP measurement used in the study, with a flatter slope in the middle of the curve, and a higher slope at both ends (y = 0.00004154x^3^–0.01115x^2^ + 1.076x – 13.34). Both the inflection point (center) of the curve, and the lower and upper limits of the autoregulation zone were obtained through the calculation of the first and second derivative of the flow curve.
**Calculation of the inflection point**:
**A.** The inflection point determining the center of the curve in the plateau region was obtained by calculating the x-intercept of the second derivative, which yielded a MAP of 89mmHg (dotted green line). This value could also be obtained from the lowest value of the first derivative (**C**). On the flow curve (**B**), this blood pressure of 89mmHg resulted in an absolute flow value of 23ml/min.
**Calculation of the autoregulation zone**:
The limits of the autoregulation zone were defined as the point on the flow curve, where the slope of the tangent to the curve reached a first derivative measurement of 0.25 indicating departure from the plateau of the curve(**D**). On the curve, this slope occurred at two locations. Through the first derivative (**C**), these two locations were determined as 52mmHg (blue) for the lower limit of the autoregulation zone and 127mmHg (red) as the upper limit. When using the original flow curve, the flow between those two boundary points of the autoregulation zone resulted in a flow range from 19±0.3 to 28±0.5ml/min, which corresponds into a %-difference in flow of -19±1% and 23±1% from the center of the autoregulatory range (23ml/min) (**E**).
**Figure B in S1 File. Absolute measurements of blood oxygen parameters**.
Absolute measurements of parameters derived from blood gas samples are displayed for each level (95 data-points). **A**. The oxygen supply (DO_2_, red) is greater than the myocardial oxygen consumption (MvO_2_, blue) for the majority of the blood pressure range, until 35mmHg, when then lines intersect. It was also at this point animals became hemodynamically unstable. This is shown where the curve crosses the X-axis in panel **B.**, which is the difference between DO_2_ and MvO_2_. **C.** displays the measurements of coronary sinus hemoglobin saturation (ScsO_2_), while **D**. shows the calculation of the oxygen surplus factor Ω. This calculation is traditionally done for mixed venous saturations and arterial oxygen saturations in a more clinically feasible and simplified way (SaO_2_/(SaO2-SvO_2_)), where Ω <2 indicates insufficient systemic perfusion, Ω 3.6–4.2 normal systemic perfusion and values >>2 sufficient perfusion. Panel **E**. displays the oxygen extraction ratio (O_2_ER) of the myocardium. The non-linear regression lines demonstrate that both measurements are fairly stable across the MAP range, until blood pressure drops to about 60mmHg, in which DO_2_-MVO_2_, ScsO_2_ and Ω drop, while O_2_ER rises.
(DOCX)

S2 File Raw data. (XLSX)
