# Supplementary material for: Relationship between myocardial oxygenation and blood pressure: Experimental validation using oxygenation-sensitive cardiovascular magnetic resonance
Source: PLoS One. 2019 Jan 16;14(1):e0210098. doi: 10.1371/journal.pone.0210098 (PMC6334913; doi:10.1371/journal.pone.0210098)
Supplement: S1 File — a. Calculations derived from blood gases and myocardial blood flow b. CMR sequences & image analysis Figure A in S1 File. Identification of the auto-regulation zone. The flow curve was generated by 55’368 data points from measurements of the left anterior descending coronary artery during manipulation of mean arterial blood pressures. A non-linear regression curve was fit to the data accounting for repeated measurements per subject. This resulting curve always demonstrates a positive slope at any MAP measurement used in the study, with a flatter slope in the middle of the curve, and a higher slope at both ends (y = 0.00004154x3–0.01115x2 + 1.076x – 13.34). Both the inflection point (center) of the curve, and the lower and upper limits of the autoregulation zone were obtained through the calculation of the first and second derivative of the flow curve. Calculation of the inflection point: A. The inflection point determining the center of the curve in the plateau region was obtained by calculating the x-intercept of the second derivative, which yielded a MAP of 89mmHg (dotted green line). This value could also be obtained from the lowest value of the first derivative (C). On the flow curve (B), this blood pressure of 89mmHg resulted in an absolute flow value of 23ml/min. Calculation of the autoregulation zone: The limits of the autoregulation zone were defined as the point on the flow curve, where the slope of the tangent to the curve reached a first derivative measurement of 0.25 indicating departure from the plateau of the curve(D). On the curve, this slope occurred at two locations. Through the first derivative (C), these two locations were determined as 52mmHg (blue) for the lower limit of the autoregulation zone and 127mmHg (red) as the upper limit. When using the original flow curve, the flow between those two boundary points of the autoregulation zone resulted in a flow range from 19±0.3 to 28±0.5ml/min, which corresponds into a %-difference in flow of -19±1% a [file pone.0210098.s001.docx]

**Supplemental Material**

***Relationship between myocardial oxygenation and blood pressure: Experimental validation using oxygenation-sensitive cardiovascular magnetic resonance***

*Dominik P. Guensch MD^1,2^, Kady Fischer PhD^1,2,3^, Christof Jung MD ^1^, Samuel Hurni MD^4^, Bernhard M. Winkler MD^4^, Bernd Jung PhD^2^, Andreas Vogt MD^1^, Balthasar Eberle MD^1^

[Supplementary methods: 2](#_Toc533069280)

[Figure A: Identification of the auto-regulation zone 3](#_Toc533069281)

[Figure B: Absolute measurements of blood oxygen parameters 5](#_Toc533069282)

# Supplementary methods in S1 File.

**Calculations derived from blood gases and myocardial blood flow**

Arterial (CaO_2_) and coronary sinus (CcsO_2_) oxygen content were calculated using hemoglobin concentration (Hb), hemoglobin saturation (SO_2_) and partial pressure of oxygen (pO_2_) from blood gas measurements: (*Hb x SO_2_ x 1,34) + (pO_2_ x 0,0031)*.

Myocardial oxygen delivery (DO_2_) was calculated as the product of arterial oxygen content and LAD blood flow (Q*_LAD_*): *CaO_2_ x Q_LAD_*. Myocardial oxygen consumption in LAD territory (MVO_2_) was derived from arterial-to-coronary sinus oxygen content difference and LAD blood flow at the time of oximetry sampling: *(CaO_2_ - CcsO_2_) x Q_LAD_*.

From these the difference in DO_2_ and MVO_2_ *(DO_2_ - MVO_2_)* was calculated.

Oxygen extraction ratio (O_2_ER) was calculated from arterial and coronary sinus oxygen contents: *100 x (CaO_2_ - CcsO_2_) / CaO_2_)*.

Oxygen excess omega (Ω) of the myocardium was derived from the same measurements using the equation *(CaO_2_)/(CaO_2_- CcsO_2_)*[1]*.*

Myocardial lactate production was assessed by calculating the difference between systemically circulating arterial (Lac_a_) and coronary sinus blood lactate (Lac_cs_) concentration: Lac_myo_=Lac_cs_-Lac_a_.

**CMR sequences & image analysis**

Left ventricular (LV) function was assessed using an ECG-gated balanced steady-state free precession (SSFP) sequence (echo time (TE) and repetition time (TR) 1.5ms and 3.4ms, respectively; flip angle 50°; voxel dimensions 1.7x1.3x6.0mm; matrix 256x173; bandwidth 977Hz/px), covering the entire left ventricle by 8-10 short-axis slices during short breath-holds (8-12s). OS-CMR imaging was performed for two short axis slices, i.e., mid-ventricular and mid-apical downstream to the blood flow probe, using an ECG-triggered SSFP sequence, (TE/TR 1.48ms/3.4ms; flip angle 50°; voxel dimensions 2.0x2.0x10.0mm; matrix 176x132, bandwidth 693Hz/px) (6-10s). All images were analyzed using clinically certified evaluation software (cvi^42^, Version 5.2, Circle CVI Inc., Calgary, Canada). For the OS images, epi- and endocardial contours were traced in end-systolic images. Signal intensities (SI) of both short-axis slices were averaged. At each MAP level, relative SI change from baseline (in percent) was calculated for to assess myocardial oxygenation changes by OS-CMR. OS-SI changes were then compared to the deflections of the other measured and calculated parameters from baseline 70mmHg. For cardiac function parameters, contours were defined in systolic and diastolic images according to standard protocol.

1. Barnea O, Santamore WP, Rossi A, Salloum E, Chien S, Austin EH. Estimation of oxygen delivery in newborns with a univentricular circulation. Circulation. 1998;98: 1407–1413.

# Figure A in S1 File. Identification of the auto-regulation zone.


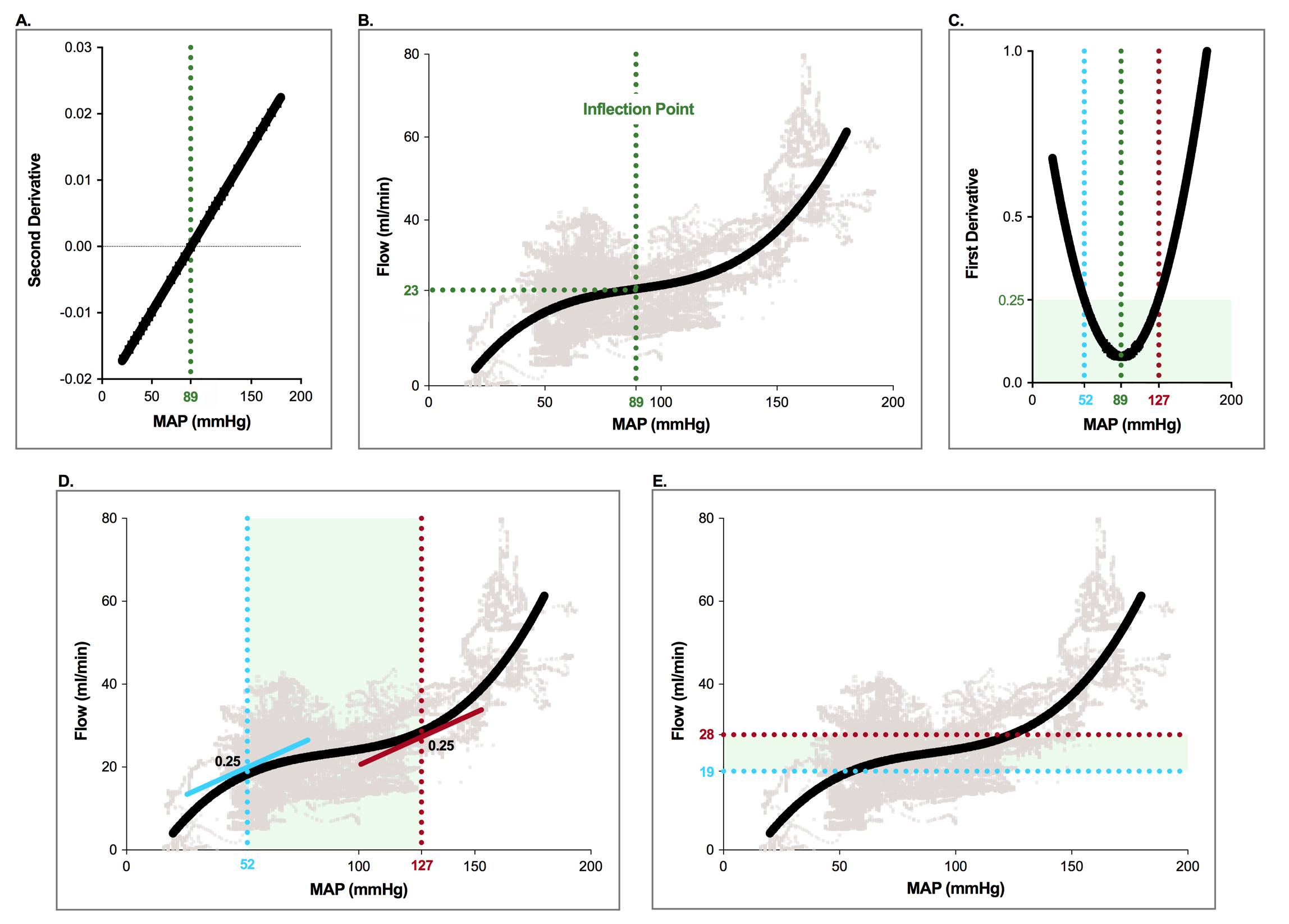


The flow curve was generated by 55’368 data points from measurements of the left anterior descending coronary artery during manipulation of mean arterial blood pressures. A non-linear regression curve was fit to the data accounting for repeated measurements per subject.

This resulting curve always demonstrates a positive slope at any MAP measurement used in the study, with a flatter slope in the middle of the curve, and a higher slope at both ends (y = 0.00004154x^3^ – 0.01115x^2^ + 1.076x – 13.34). Both the inflection point (center) of the curve, and the lower and upper limits of the autoregulation zone were obtained through the calculation of the first and second derivative of the flow curve.

**Calculation of the inflection point:**

**A.** The inflection point determining the center of the curve in the plateau region was obtained by calculating the x-intercept of the second derivative, which yielded a MAP of 89mmHg (dotted green line). This value could also be obtained from the lowest value of the first derivative (**C**). On the flow curve (**B**), this blood pressure of 89mmHg resulted in an absolute flow value of 23ml/min.

**Calculation of the autoregulation zone:**

The limits of the autoregulation zone were defined as the point on the flow curve, where the slope of the tangent to the curve reached a first derivative measurement of 0.25 indicating departure from the plateau of the curve(**D**). On the curve, this slope occurred at two locations. Through the first derivative (**C**), these two locations were determined as 52mmHg (blue) for the lower limit of the autoregulation zone and 127mmHg (red) as the upper limit. When using the original flow curve, the flow between those two boundary points of the autoregulation zone resulted in a flow range from 19±0.3 to 28±0.5ml/min, which corresponds into a %-difference in flow of -19±1% and 23±1% from the center of the autoregulatory range (23ml/min) (**E**).

# Figure B in S1 File. Absolute measurements of blood oxygen parameters.


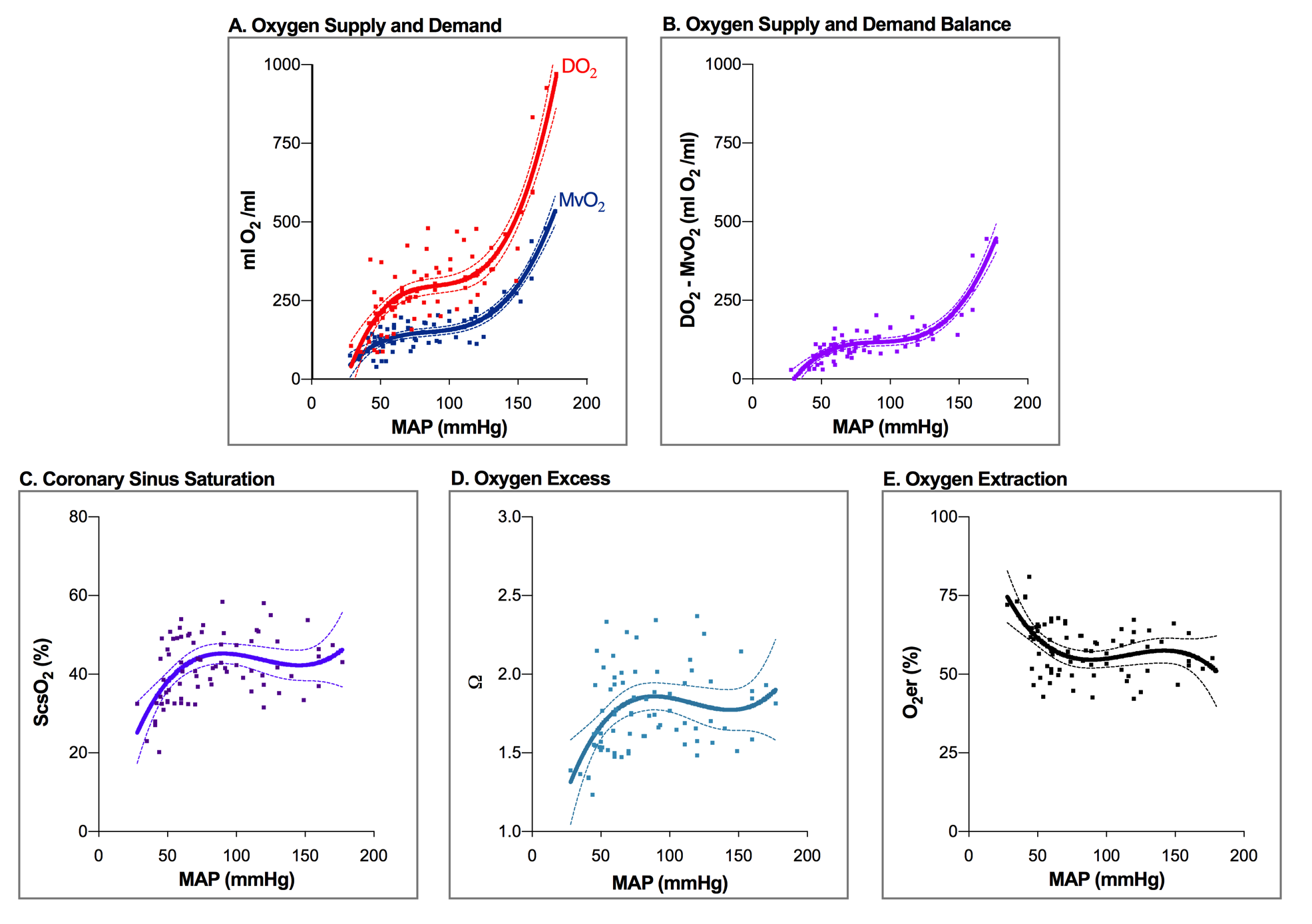


Absolute measurements of parameters derived from blood gas samples are displayed for each level (95 data-points). **A**. The oxygen supply (DO_2_, red) is greater than the myocardial oxygen consumption (MvO_2_, blue) for the majority of the blood pressure range, until 35mmHg, when then lines intersect. It was also at this point animals became hemodynamically unstable. This is shown where the curve crosses the X-axis in panel **B.**, which is the difference between DO_2_ and MvO_2_.

**C.** displays the measurements of coronary sinus hemoglobin saturation (ScsO_2_), while **D**. shows the calculation of the oxygen surplus factor Ω. This calculation is traditionally done for mixed venous saturations and arterial oxygen saturations in a more clinically feasible and simplified way (SaO_2_/(SaO2-SvO_2_)), where Ω <2 indicates insufficient systemic perfusion, Ω 3.6-4.2 normal systemic perfusion and values >>2 sufficient perfusion.

Panel **E**. displays the oxygen extraction ratio (O_2_ER) of the myocardium. The non-linear regression lines demonstrate that both measurements are fairly stable across the MAP range, until blood pressure drops to about 60mmHg, in which DO_2_-MVO_2_, ScsO_2_ and Ω drop, while O_2_ER rises.
